# Food Bioactive Compounds against Diseases of the 21st Century

**DOI:** 10.1155/2015/241014

**Published:** 2015-02-22

**Authors:** Chia-Chien Hsieh, Juliana Maria Leite Nobrega de Moura Bell, Blanca Hernández-Ledesma

**Affiliations:** ^1^Department of Human Development and Family Studies (Nutritional Science & Education), National Taiwan Normal University, No. 162, Section 1, Heping East Road, Taipei 106, Taiwan; ^2^Food Science Department, University of California, Davis, 631 Hilgard Lane, Davis, CA 95616, USA; ^3^Instituto de Investigación en Ciencias de la Alimentación (CIAL, CSIC-UAM, CEI UAM+CSIC), Nicolás Cabrera 9, 28049 Madrid, Spain

Increasing incidence of metabolic disorders has been evidenced in the past decades. Common metabolic disorders include obesity, diabetes, metabolic syndrome, and other chronic diseases, including cardiovascular and neurological diseases, stroke, cancers, immune disorder, and chronic respiratory diseases. Despite clear evidence of the impacts of those diseases on human health, their incidence is reaching epidemic proportions in industrialized countries, with similar trend being observed in developing countries. Understanding genetic and environmental factors that might promote those diseases became a crucial research topic. Diet and lifestyle are two environmental factors having a significant impact on those diseases. Thus, modifications of diet and lifestyle are becoming a new strategy for prevention/treatment of metabolic disorders. Foods contain a wide range of bioactive compounds with multiple physiological properties. This research area has increased in the last years, with growing numbers and a wide variety of functional foods available in the worldwide market. Because of the importance of a healthy diet on metabolic disorders prevention, the present special issue summarizes the most recent advances on applied science to foods considered as a source of bioactive compounds that could be potentially useful to prevent/treat different diseases of the 21st century. The selected papers represent a rich and many-facet knowledge, which we have the pleasure of sharing with the readers.

Potential health benefits of naturally occurring *α*-linolenic acid have been described in a review entitled “*α*-Linolenic Acid, an Omega-3 Fatty Acid with Neuroprotective Properties: Ready for Use in the Stroke Clinic?” In this review, N. Blondeau et al. highlighted some protective effects of *α*-linolenic acid on neurological disorders and possible mechanisms of action. Sources of naturally occurring *α*-linoleic acid, intake recommendations, and current state of* in vitro* and* in vivo* tests have been reported.

In a paper entitled “Antioxidant/Prooxidant and Antibacterial/Probacterial Effects of a Grape Seed Extract in Complex with Lipoxygenase” by V. S. Chedea et al. The biological activities of flavan-3-ols and procyanidins from grape seeds, pure catechin, and an aqueous grape seed extract were evaluated in the presence of lipoxygenase or in extract. Those fractions were applied to leucocyte culture,* Escherichia coli B41* and* Brevibacterium linens* where lipid peroxidation, cytotoxicity, and growth rate of exposed cells were evaluated.

In a paper entitled “Role of Feed Forward Neural Networks Coupled with Genetic Algorithm in Capitalizing of Intracellular Alpha-Galactosidase Production by* Acinetobacter sp.*” S. Edupuganti et al. enhanced the production of intracellular alpha-galactosidase (7.5 to 10.2 U/mL) from* Acinetobacter sp.* isolated from sugar cane waste by using hybrid artificial neural networks and genetic algorithm (ANN-GA).

In a paper entitled “Angiotensin I Converting Enzyme Inhibitory Peptides Obtained after* in Vitro* Hydrolysis of Pea* (Pisum sativum var. Bajka) *globulins” A. Jakubczyk and B. Baraniak evaluated the potential antihypertensive effect of peptides released from pea seed globulins under conditions simulating gastrointestinal digestion. These peptides are demonstrated to act as uncompetitive inhibitors of angiotensin-converting enzyme activity.

In a paper entitled “Anti-Inflammatory Effects of* Siegesbeckia orientalis *Ethanol Extract in* in Vitro* and* in Vivo* Models” Y.-H. Hong et al. demonstrated that* Siegesbeckia orientalis* ethanol extract attenuated local and systemic acute inflammation in both* in vitro* and* in vivo* studies by inhibiting inflammatory mediators through suppression of MAPKs and NF-*κ*B dependent pathways.

In a paper entitled “Evaluation of the Antioxidant Activity and Antiproliferative Effect of the* Jaboticaba (Myrciaria cauliflora) *Seed Extracts in Oral Carcinoma Cells” C.-G. Lin et al. pointed to the potential of* Jaboticaba* seed extract as a chemopreventive agent against oral carcinoma cells. The antioxidant activity and the apoptosis-inducing properties are responsible for the observed effects.

In a paper entitled “The Study of Interactions between Active Compounds of Coffee and Willow* (Salix sp.) *Bark Water Extract” A. Durak and U. Gawlik-Dziki demonstrated that both coffee and willow bark are sources of multidirectional antioxidant compounds. The extracts from willow as an ingredient in coffee beverages can provide health promoting may be benefit to consumers.

In a paper entitled “Oral and Intraperitoneal Administration of Quercetin Decreased Lymphocyte DNA Damage and Plasma Lipid Peroxidation Induced by TSA* in Vivo*” S.-T. Chan et al. pointed quercetin administered intraperitoneally decreased trichostatin A-induced lymphocyte DNA damage and plasma lipid peroxidation, but no effect on tumor growth was observed, indicating the potentially auxiliary protection and used pathway of quercetin in chemotherapy.

In a paper entitled “Food-Derived Bioactive Peptides on Inflammation and Oxidative Stress” F. Jahandideh and J. Wu showed the roles of various food-derived bioactive peptides in inflammation and oxidative stress and discussed the potential benefits and limitations of using these compounds against the burden of chronic diseases.

In a paper entitled “Milk Proteins, Peptides, and Oligosaccharides: Effects against the 21st Century Disorders” C.-C. Hsieh et al. summarized the impact of proteins, derived-peptides, and oligosaccharides present in milk on human health, with special emphasis on their effects against most common chronic disorders nowadays.

